# The Pandemic and Changes in the Self-Perception of Teacher Digital Competences of Infant Grade Students: A Cross Sectional Study

**DOI:** 10.3390/ijerph18094756

**Published:** 2021-04-29

**Authors:** Rosalía Romero-Tena, Carmen Llorente-Cejudo, María Puig-Gutiérrez, Raquel Barragán-Sánchez

**Affiliations:** 1 Department of Teaching and Educational Organization, University of Seville, 41013 Seville, Spain; rromero@us.es (R.R.-T.); rbarragan@us.es (R.B.-S.); 2 Department of Didactics of Experimental and Social Sciences, University of Seville, 41013 Seville, Spain; mpuig@us.es

**Keywords:** COVID, digital competences, learning, students, university, self-perception, educational challenges

## Abstract

Without having a reaction time, the pandemic has caused an unprecedented transformation in universities around the world, leading to a revolution from structured models anchored in the conception of transmission of training towards a teaching approach-learning saved thanks to the incorporation of technology. This study aims to verify whether the pandemic situation has influenced the digital competence self-perception of students. Comparing two groups during the academic years 2019/2020 and 2020/2021, the instrument used is the questionnaire for digital competence “DigCompEdu Check-In” for future teachers. After the educational intervention, group A (before COVID-19) presented higher self-perceptions of competence than group B (during COVID-19); the pandemic situation caused by COVID-19 has negatively influenced students’ self-perception of their digital skills in the pretest in the different dimensions under study. Before receiving the training, the group that did not experience the pandemic enjoyed a higher self-perception of their competencies than the group that experienced the pandemic. The data obtained indicate that the difference exists, and that it is statistically significant, and may be a consequence of the clear relationship between self-perception and the way in which students face reality through their personal and subjective vision.

## 1. Introduction

Due to exceptional situations, great transformations have taken place in different contexts; the university, like others, pushed to adapt to circumstances for which it was not prepared for, improvising actions that at this point, it would be convenient to assess. Without a reaction time, the COVID-19 pandemic caused an unprecedented transformation in universities around the world, leading to a revolution from structured models anchored in the conception of transmission of training and in person as basic pillars of teaching between teachers and students, towards a teaching-learning approach saved thanks to the incorporation of technology and virtual training platforms (a learning management system). Following concerns about this changed, which has modified our actions but was barely considered in the haste to solve the problem that has arisen, this article aims to show the impact of the pandemic on the development of teacher digital competences of university students through the Infant Education Degree, which provides essential competencies for their professional development as future teachers.

### 1.1. Impact of COVID on University Education

Part of the academic years 2019/20 and 2020/21 will be remembered in history for the fact that more than 1500 million students throughout the world were forced to not attend their classrooms after lockdowns was imposed to contain the spread of the SARS-Cov-2 virus, which causes COVID-19. In this way, the face-to-face modality gave rise, from one day to the next, to the so-called online, telematic or virtual modality [1].

For a long time, several questions were raised regarding the penetration of technologies in teaching. Firstly, beliefs teachers have about possibilities that technologies can offer for their professional development are elementary, both for incorporating them properly, and for the form or method used to do so [2,3,4,5,6]. Specifically, [7] (p. 339) point out that: “Teachers’ beliefs about learning and teaching are propositions about learning and teaching that a teacher holds to be true; they develop over the many years that teachers spend in school, first as students, then as teachers, and with time and use, these beliefs become robust.”

Another issue is that there is a tendency among teachers to conceive of technologies simply as an addition to their entire teaching process, but not as tools that can facilitate a true educational revolution, a change and a process of authentic innovation with which to build new ways to learn [8,9].

Among this mass of issues, COVID-19 has arisen, causing its greatest radical change in higher education. It meant a drastic break with many of the traditional approaches, such as the unity of space and time, and unity of action.

In Spain, as other authors point out [10], unlike in Latin America, the lack of learning technologies has been not the greatest barrier to coping with this situation [11] (p. 85). The problem has been revealed in people, as many of those involved have reported skills deficiencies in the use of these technologies, problems integrating them into the instructional design of their subjects or simply ignorance about institutional technological solutions that universities provided at their disposal. 

This is the time, therefore, to offer the possibility of verifying how the digital transformation is taking place in universities, focusing on the training of university students and, therefore, on the necessary training in digital teaching competencies of the teachers [9,12,13].

In this context, it is possible to establish a direct relationship between the appearance of the pandemic with the physical health of university teachers and students and their academic development during this school year. Reference [14] claims that the virus is the condition that has prompted this mental leap. As a result of its spread, students must adopt coping strategies that are very different from those they had until now, before entering the university, to successfully overcome the new demands that are demanded of them. As [15] (p. 5) points out, “a large part of these new university students lack these strategies or present academic behaviours that are inappropriate for the new demands. Entering a university (with the changes it entails) represents a set of highly stressful situations due to the fact that the individual may experience, even if only temporarily, a lack of control over the new environment, potentially generating stress and, ultimately term, potential generator-with other factors-of university academic failure”.

If university life was a particularly stressful period, today young people must get used to the new educational context and the form of learning (online) required to stop infections during the pandemic; these are novel experiences that can cause confusion in students, especially when developing their digital skills and personal skills. With the exception of some studies, particularly developed in China [12,16,17] there are still few assessments of the psychological effects that the pandemic has caused on the mental health of university students, which were already defined as a vulnerable population.

Therefore, it would be interesting to assess the effects of the pandemic not only on mental health and personal well-being but also academically. Even more so, special emphasis can be placed on the gender variable as a determinant of these possible effects, if it takes into account that different international studies show important gender inequalities, since “it is women who report and are diagnosed more frequently of some problem of this kind. This reality, however, is more complex when analysed according to the different mental disorders (…)” [18] (p. 61).

Problems of anxiety, stress, etc., are the main impediments to success in education, since they can affect students’ motivation, concentration and social interactions. In our study, it is important to reconsider that having digital skills can also influence these problems; the new environments imposed by the pandemic have been able to alter the academic habits of many female students, causing anxiety about an immediate adaptation to the new system. 

### 1.2. Student Self-Perception

According to the above, and focusing on the way in which learning develops in university students, different studies [19] show how this is clearly related to self-perception and the way in which they face reality through the personal and subjective vision of their own image.

We understand self-perception as the valuations that a person has of himself, in a field of action and a specific moment, as well as the “set of beliefs, attitudes, desires, values and expectations of the outside world and that the individual transforms in his inside world” [20] (pp. 96–97).

Therefore, we consider that when an individual learns, they perform a self-regulation process which is directly influenced by the evaluation he makes of his own competences [19,21].

In this way, various studies [22,23,24,25] show there is a direct relationship between learning and the way the individual feels about himself and is perceived as being competent, hence the importance of analyzing emotions in the learning process. In this context, the study carried out by [26] concludes that students manifest a diversity of both positive and negative emotions in learning processes supported in virtual environments, finding higher scores in positive emotions than in negative ones. As was already pointed out, according to [27], factors such as anxiety, worries and discomfort hinder learning. This type of situation, which directly affects the way the world is perceived and the role that one plays in it, means that students do not perceive all the nuances of the information and fail to process it properly, a fact that ends up re-impacting on their academic performance.

In addition to all this, another concept that is closely related to self-perception, when we refer to learning, is that of self-efficacy. The concept of self-efficacy comes from Bandura’s social cognitive theory [28] and can be defined as the perception that the individual himself generates about his capacities to face future situations. According to Bandura [28] self-efficacy derives from four main sources: experiences or achievements of mastery, vicarious experiences, verbal persuasion and physiological and affective states or emotional arousal. The first refers to the experiences in which the individual must face the performance of the task, and therefore requires implementation and according to the author is [29] (p. 71) the most influential source in the configuration of self-efficacy “The most effective way of creating a strong sense of efficacy is through mastery experiences. Successes build a robust belief in one’s personal efficacy. Failures undermine it, especially if failures occur before a sense of efficacy is firmly established”. Vicarious experiences allude to observational learning, while modeling and social persuasion are related to exposure to comments and verbal expressions which an individual is subjected to at the time they perform some action. Finally, the physiological and affective states or emotional arousal refers to the emotional state, pointing out in the words of Bandura [28] (p. 198): “Because high arousal usually debilitates performance, individuals are more likely to expect success when they are not beset by aversive arousal than if they are tense and viscerally agitated”. In relation to all this, various studies have been developed that show the relationship between moods and psychological well-being with self-efficacy [30,31]. Others establish a relationship between self-efficacy and academic performance, indicating that self-efficacy is a key element in performance and skills development [32,33].

From these considerations, it is interesting to address the self-perception of future teachers’ learning in terms of digital competence, in a context of a health crisis.

### 1.3. Development of the Teacher Digital Competences (TDC) of Future University Teachers

The study presented in this article has as its starting point the key competences defined by the Council of the European Union [34], the so-called DIGCOM 2.0. This is the most widely used competence framework for the development and understanding of digital competence in Europe [35], and educational institutions use it to train people capable of integrating technologies into their daily lives in a profitable, safe and healthy way.

If we want to ensure our future university teachers are digitally trained according to the proposed European Framework for Digital Competence of Teachers “DigCompEdu” [36], we will have to take into account different areas of competence they must acquire, specifically we are talking about: (A) Professional commitment: their ability to use digital technologies not only to improve teaching, but also to interact professionally with colleagues, students, family and different agents of the educational community. (B) Digital resources: any teacher must develop and identify good educational resources, being able to modify, create and share them to adjust them to their objectives, students and teaching style. At the same time, you must know how to use and manage digital content responsibly, respecting copyright rules and protecting personal data. (C) Digital pedagogy: this involves knowing how to design, plan and implement the use of digital technologies in the different stages of the teaching and learning process immersed in methodologies that are centered on the student body. (D) Evaluation and feedback: linked to the use of digital tools and strategies in the evaluation and improvement of teaching-learning processes. (E) Empower students: the use of digital tools to promote the active participation of students in the learning process and their autonomy over it. (F) Facilitate students’ digital competence and assess how to develop and facilitate citizens’ digital competence [37]. Likewise, to seek self-perceptions of their digital skills in students, “DigCompEdu Check-In” was designed for future teachers’ [38] adaptation of the “DigCompEdu Check-In” for teachers [39], which we referred to and which we will describe in Section 2.3.

As a result of all the above, the question that arises in the study is to verify if the pandemic (COVID-19) affected the development of TDC of the students of the Infant Education Degree, after they received technological training for their professional development. 

## 2. Materials and Methods

We decided to carry out an inferential analysis to check if the pandemic situation influenced the students’ self-perceptions of competence. Two groups were compared: group A that took the subject of Information and Communication Technology Applied to Early Childhood Education before the pandemic, and group B that took this same subject during the pandemic period.

### 2.1. Objectives 

The main objective was to determine the impact caused by COVID-19 on the development of TDC in Infant Grade students to draw up plans that could help alleviate this impact. To deepen this objective, it was specified through specific objectives. The first is checking the reliability and validity of the instrument used to measure the level of self-perception of the TDC (O1), then describing the results obtained in the competence self-perception for group A and group B (O2), then checking whether there are statistically significant differences between the results obtained in both groups (O3). Finally, checking if there is a relationship between competency self-perception and the variables sex, teaching experience and time of use of the technology (O4).

### 2.2. Sample

The population that makes up the study sample was made up of students who are studying the 4th year of a Bachelor’s Degree in Early Childhood Education at the University of Seville, during the academic years 2019/2020 and 2020/2021, who have taken the subject of ICT applied to Infant Education (TICEI) in the first semester. The sample was made up of a total of 559 students: From the 2019–2020 academic year, 296 students participated (group A) and from 2020–2021 a total of 263 participated (group B). Group A was the one that received technological training before the pandemic and group B received technological training during the pandemic. For their selection, incidental or convenience criteria were chosen according to their availability to answer the questionnaire [40]. It should be pointed out that both groups received the same training and in both a pretest (f = 232) and a posttest (f = 237) of the DigComEdu for students [38] were administered.

The characteristics of both groups of subjects (group A and group B) were similar, since most of the students are women, being 92.6% in group A and 95.6% in group B. Many of them are in the 20–25 age bracket (75.8% in group A and 88.4% in group B). We would like to highlight that there is a significant percentage of students who have already had teaching experience: 39% in Group A and 22% in Group B. A similar percentage of the sample said that they used ICT as an educational tool for 3 years (A = 25.3% and B = 25.9%), followed by those with less than 1 year (A = 25.6% and B = 15.7%) and those who never used it (A = 13% and B = 11.7%).

There were considerable differences between both groups in terms of the time dedicated to the use of technology for their studies, as group A (before the pandemic) indicated a usage rate of 51–75% while group B provided a rate of 76–100%, respectively. When asked about the use of technology for their careers, group A’s response was 51–75% of the time while group B’s response was 76–100%, which may have been caused by the lockdown situation and the virtual nature of classes after the pandemic began. Finally, it is necessary to highlight that in both groups, a high percentage of the sample (group A = 89.4; group B = 84%) was interested in learning to use new applications and digital resources. 

A link was added to the training program so that the aspects related to the training received for both groups can be consulted: https://n9.cl/z1qnc (accessed on 24 February 2021).

### 2.3. Instrument

The instrument used was the “DigCompEdu Check-In” questionnaire for digital competence for future teachers [38] (see Table 1). It is an adaptation made for students (see Table S1) and it has “DigCom-pEdu Check-In” as a starting point for teachers [39]. It arises from a process of expert consultations, tests prior to the pilot phase and a review of elements [41]. In the adaptation, the structure of origin has been respected, where each competence is represented by a single item and by selecting the most generic concept that encompasses all the specific content of the competence. The 22 items that made up the questionnaire respond to the 6 competence areas: professional commitment (4), digital resources (3), digital pedagogy (4), evaluation and feedback (3), empowering students (3) and facilitating competition digital students (5). Each item was measured on a 5-interval Likert scale, where participants indicate the extent to which they reflect their own assessment, selecting one of the five options. The instrument includes a final section in which the sociodemographic data of the students and some questions about their activities, habits, etc. are collected. The questions are organized progressively, reflecting the general progression logic of “DigCompE-du” (proficiency levels), through an internal scoring system. Six progressive competence levels were assessed so that the level of digital competence of a teacher is identified, from the Novice level (A1) or one with very little experience and contact with educational technology, to the Pioneer (C2) or one who leads innovation with ICT [37]. Once the modifications were made, a pilot study was carried out with Infant and Primary Grade students, during which time after validating this version by 256 students, the Cronbach’s alpha coefficient was 0.947 [38].

In addition, statistical tests were carried out that allow us to complement the reliability and validity with our sample under study (O1).

Next, results achieved on the reliability obtained for the total construct and for its dimensions are presented; to achieve this, Cronbach’s Alpha and McDonald’s Omega were used.

For some authors [42,43], the Alpha and Omega values situated between the interval 0.8 and 1 can be considered ‘very high’. Likewise, it is considered that values greater than 0.7 are sufficient to guarantee the reliability of the instrument. Consequently, they would denote high levels of reliability both for the globality and for the different dimensions that make up the instrument.

To check the validity, the coefficients of Composite Reliability (CR), Average Extracted Variance (AVE) and Maximum Shared Variance (MSV) were calculated. Table 2 shows the results, as well as the reference values taken for the model fit [44].

All the figures obtained were adjusted with the reference values (CR> 0.7, AVE > 0.5 y MSV < AVE). Therefore, the reliability of the model (CR) as well as its convergent (AVE) and discriminatory (MSV) validity is demonstrated.

### 2.4. Data Analysis Procedure 

The work proposed a pretest—posttest methodology through a descriptive cross-sectional study. This design did not modify the variables under study, but explored their nature and behaviour in the participants who were part of the study

Descriptive and central tendency (O2) analysis was carried out. In addition, contrast statistics were applied to make comparisons between the scores obtained in group A (before the pandemic) and group B (during the pandemic) (O3). Specifically, the non-parametric Mann–Whitney U test was used. Additionally, to test hypotheses, the correlational analysis method between items and dimensions (O4) was used. To do this, the bivariate correlational analysis technique was used by means of Spearman’s Rho correlation coefficient. In parallel, it was proven that the data are not normally distributed through the study of skewness and kurtosis. The Kolmogorov–Smirnov goodness of fit test confirmed this verification with a significance (*p*-value) equal to 0.000 for all items (a non-normal distribution). At all times, the data obtained were analyzed with the SPSS statistical package (v.23).

## 3. Results

Following the objectives of the study, data showing the competence areas and the degree of self-perception of the students before and after taking the subject “Information and Communication Technologies Applied to Early Childhood Education” in group A and group B (O2) were assessed.

Table 3 presents the results obtained in the mean and standard deviation by the groups (A and B) study dimension.

As can be seen, before the educational intervention, group A (before COVID-19) presented higher self-perceptions of competence than group B (during COVID-19); On the other hand, after the educational intervention, self-perceptions tended to equalize, although they were still higher in group A.

To check whether the changes detected in competence self-perceptions between group A and group B were statistically significant changes, the non-parametric Mann–Whitney U contrast tests were applied for more than two related samples (O3) (Table 4).

According to the data obtained, in general, the pandemic situation caused by COVID-19 has negatively influenced students’ self-perception of their digital skills in the pretest for the different dimensions under study. Almost all competency areas showed a significance level lower than 0.05, so we can confirm, with a 99% confidence level, that there were statistically changes between groups A (before COVID-19) and group B (during the COVID-19 pandemic). Similarly, it is necessary to indicate that there were no significant differences in area 4 related to the evaluation.

If Table 5 is observed, where the average ranges are shown, the changes produced always imply an improvement in the self-perception of the students who have taken the subject before COVID-19, (Group A) except in dimension or area 4.

In the same way, we verified whether the differences obtained in the self-perceptions produced between the two groups for the posttest are significant (Table 6). The data showed that almost all the competence areas presented a level of significance lower than 0.05, so we could confirm, with a 99% confidence level, that there are statistically significant changes between before COVID-19 and during COVID-19 in the competence self-perception for the posttest. Similarly, it is necessary to indicate that there were no significant differences in area 4 related to the evaluation.

As in the analysis carried out for perceptions in the pretest, the ranges showed (Table 7) an improvement for group A, although after training there was a tendency to equalize that could be linked to the training received and technological use after training while taking the subject.

Finally, a correlational analysis was carried out to check if there is a relationship between the variable “Time” using technology and the competence self-perception of each area, as well as the general perception of digital competence. For this, the Spearman Rho correlation coefficient was applied in a comparative way in group A (before the pandemic) and group B (during the pandemic) (O4). The results are shown in Table 8.

As can be seen, in group A (before the pandemic) there was no relationship between the variables time of use of ICT and self-perception of DC in all of its areas. On the other hand, in group B with a confidence level of 99%, it could be affirmed that there is a positive correlation, although of low intensity, which could be explained by the alarm situation and the confirmation that this group made greater use of ICT as an educational tool, which positively influenced their competence self-assessment of themselves.

In a complementary way, it was verified whether there is a relationship between competency self-perceptions and the variables sex and teaching experience. Regarding the gender variable, the data showed a positive relationship between the female gender and positive self-perceptions, but the sample is mostly female, so we decided to continue focusing on this variable when the sample is expanded. Regarding the relationship between the variable teaching experience and competence self-assessment, no statistical evidence was found that there is a clear relationship between both variables.

## 4. Discussion and Conclusions

The harsh impacts generated by lockdowns on the state of the digital transformation of universities has been significant, either because the expectations that were placed on ICT were too high or because the incorporation of ICT had never been projected from a true conceptual pedagogical base that supports the real possibilities that these ICT opportunities present. What is clear is that a global pandemic has managed to highlight its most positive side, and thus makes many institutions aware of the distance between their development and their strategic planning in this regard. Of all the dimensions of this digital transformation, if anything has been learned, it is that there is an unequivocal need for digital training for teachers and students.

University students are part of a population that is considered particularly vulnerable to external problems that are likely to take a toll on their mental health. The results of this study show the effects that the abrupt transition to pandemic conditions has had on self-perceptions of their TDC, all of which could be linked to possible mental health problems, anxiety or stress as possible causes. In the study carried out, it could be observed how they suggest the existence of a considerable negative impact of the COVID-19 pandemic on a variety of academic aspects that influence lifestyles [42].

The findings obtained on the influence of the pandemic on the self-perception of GI students revealed that, before receiving the training, the group that did not experience the pandemic enjoyed a higher self-perception of their skills than the group that experienced the pandemic. In a later measurement (posttest), results continue to be more positive in the perceptions of the group not in a situation of alarm compared to the one in that situation, although the trend has been to equalize, perhaps due to the formative effect of the subject studied. It has already been pointed out that self-perception is marked by the set of external aspects that each individual transforms into their inner world [20]. On this occasion, it is clear that there is an extraordinary situation worldwide as a possible cause of some inequality between the two groups. The data shown in the study indicate that the difference exists and that it is statistically significant, and may be a consequence of the clear relationship that exists between self-perception and the way in which students face reality through personal vision and the subjectivity of experiences [19], just as it is known that there is a direct relationship between learning and the way in which the individual feels about himself and is self-perceived as being competent [22,23,24,25]. Hence, factors such as anxiety, worries and discomfort make learning difficult by causing students not to perceive all the nuances of information and fail to process it properly, a fact that ends up having an impact on their academic performance [27].

At the same time, a clear positive relationship between the time of use of technology and a greater self-perception of competence due to the change in academic situation caused by the pandemic has been verified, which generated the evident need for a greater use of ICT as an educational tool and the only option to continue with their training, thus marking a clear difference between the group prior to COVID and the one that was formed during COVID. All this makes sense under the theories that show that self-efficacy is influenced by four factors: experiences or achievements of mastery, vicarious experiences, verbal persuasion and physiological and affective states or emotional arousal [28].

Another of the study’s conclusions is that the results of the different analyses carried out corroborated that the DigCompEdu Check-In instrument, adapted to the Spanish context and used with university students during COVID-19, can be presented as a valid and reliable instrument to measure DTC. Consequently, its use is feasible, given its psychometric properties (O1). Likewise, it must be taken into account that confidence is presented as a self-perception, reaffirming the data provided by [38].

That there is a direct relationship between the self-perception of the students about their TDC and the pandemic (O2), or that gender may be an influencing variable in this case, cannot be assumed due to the lack of representation of the male gender in the classrooms of early childhood education. It also cannot even be considered as a faithful reflection of the reality in which this educational level unfolds. Furthermore, as they are forced to be absent, the students have used the technologies for longer, influencing their increase in the self-perception of their TDC after training.

This study shows that the effects of the pandemic that have been experienced—and continue to be suffered—go beyond purely academic aspects such as grades. The effects of isolation, social distancing and fear of suffering from the disease are causing changes in our way of perceiving the environment that surrounds us and ourselves, which can lead to negative learning outcomes. Education plays a fundamental role as a backbone for the acquisition of fundamental digital competences at the present time, so it is necessary to rethink the study plans seeking a greater adaptation to the demands in TDC that the European framework proposes. We must take advantage of the situation and go a step further, without conceiving training as a mere technological adoption in an unexpected way, but rather that everything that happened provides the bases and foundations to structure training plans where TDC are contemplated in an equitable manner with tools, resources, platforms, etc. [45] What is needed is a more effective, more open and flexible model, with diversified training formats, with adequate training and with resources that facilitate the acquisition of skills, in short, with a reflection of the entire university community involved in achieving a true process of digital transformation [46].

It is clear that 2020 will be remembered as the year that made human beings think about their vulnerability and the uncertainty of the future. This new reality showed the role that must be interpreted through training and action. Perhaps this can occur with the hope of a moment of reflection that allows us to recognize ourselves and reconnect with nature and with “the other”, in an agreement in which life is revalued and where solidarity and compassion towards oneself and towards others be the axes of the compass that give direction to the new 21st century. 

Finally, it is important to point out that it is necessary for universities to plan actions that mitigate the effects of the pandemic crisis for the benefit of the mental health of their students, helping them to solve their fundamental problems and taking charge, in part, of their own social impacts. In this context, the results converge on the rise in mental health problems among university students, although these contributing factors may not necessarily be generalizable to the student population of other countries.

## Figures and Tables

**Table 1:** Reliability coefficients.

Dimension	α	Ω
Professional commitment	0.832	0.851
Digital resources	0.824	0.835
Digital pedagogy	0.811	0.832
Evaluation and feedback	0.766	0.783
Empower students	0.789	0.793
Facilitate the students’ digital competence	0.882	0.885
TOTAL	0.939	0.936

**Table 2:** Convergent and discriminant validity of the model.

Dimension	CR	Adjustment	AVE	Adjustment	MSV	Adjustment
Professional commitment	0.843	CR > 0.7	0.568	AVE > 0.5	0.412	MSV < AVE
Digital resources	0.825		0.585		0.361	
Digital pedagogy	0.801		0.692		0.383	
Evaluation and feedback	0.785		0.721		0.413	
Empower students	0.772		0.670		0.428	
Facilitate the students’ digital competence	0.891		0.690		0.435	

Composite Reliability (sometimes called construct reliability) is a measure of internal consistency in scale items; Average Variance Extracted (AVE) is a measure of the amount of variance that is captured by a construct in relation to the amount of variance due to measurement error; Maximum Shared Variance (MSV) is the square of the highest correlation coefficient between latent constructs.

**Table 3:** Mean and standard deviation by groups and dimensions.

	NON_COVID		COVID		NON_COVID		COVID	
	PRETEST		PRETEST		POSTEST		POSTEST	
	M	SD	M	DT	M	DT	M	DT
A1. Professional commitment	2.17	0.62	1.94	0.62	1.97	0.67	1.83	0.59
A2. Digital resources	2.49	0.68	2.23	0.65	2.21	0.72	2.22	0.7
A3. Digital pedagogy	2.86	0.71	2.59	0.64	2.49	0.84	2.46	0.69
A4. Evaluation	2.43	0.8	2.25	0.78	2.18	0.85	2.24	0.84
A5. Empower	2.97	0.84	2.64	0.86	2.5	0.98	2.56	0.88
A6. Facilitate DC	2.49	0.83	2.48	0.77	2.05	0.94	2.34	0.81
General	2.57	0.61	2.36	0.54	2.23	0.7	2.28	0.59

M (Mean); SD (Standard Desviation), DC (Digital Competence).

**Table 4:** Mann–Whitney U test with non-COVID-19 grouping variables (pretest).

Mann–Whitney U Test							
	A1.	A2.	A3.	A4.	A5.	A6.	General
U de Mann–Whitney	10,420.000	10,146.000	9877.000	11,783.000	10,268.500	13,265.000	10,218.000
W de Wilcoxon	24,616.000	24,342.000	24,073.000	25,979.000	24,464.500	27,461.000	24,414.000
Z	−3.465	−3.800	−4.101	−1.857	−3.641	−0.107	−3.672
Sig. Asymptotic (bilateral)	0.001	0.000	0.000	0.063	0.000	0.005	0.000

Grouping variable: Group (not COVID-19 and COVID-19); A1. Professional commitment; A2. Digital resources; A3. Digital pedagogy; A4. Evaluation; A5. Empower; A6. Facilitate.

**Table 5:** Average ranges by dimensions in the pretest.

Dimension	COVID-19/Non COVID-19	Average Range
A1. Professional_commitment	COVID-19	182.47
	No COVID-19	146.52
A2. Digital_Resources	COVID-19	184.19
	No COVID-19	144.89
A3. Digital_Pedagogy	COVID-19	185.88
	No COVID-19	143.29
A4. Evaluation	COVID-19	173.89
	No COVID-19	154.64
A5. Empower	COVID-19	183.42
	No COVID-19	145.62
A6. Facilitate_CD	COVID-19	174.57
	No COVID-19	163.46
General	COVID-19	183.74
	No COVID-19	145.32

**Table 6:** Mann–Whitney U Test with a non- COVID-19– COVID-19grouping variable (posttest).

Mann–Whitney U Test							
	A1.	A2.	A3.	A4.	A5.	A6.	General
Mann–Whitney U Test	5697.000	6485.500	6250.500	6157.500	6381.500	5249.000	6331.000
W de Wilcoxon	10,257.000	15,938.500	10,810.500	15,610.500	15,834.500	14,702.000	15,784.000
Z	−1.625	−0.044	−0.514	−0.703	−0.252	−2.509	−0.351
Sig. Asymptotic (bilateral)	0.005	0.001	0.005	0.010	0.000	0.001	0.000

Grouping variable: Group (not COVID-19 and COVID-19).

**Table 7:** Average ranges by dimensions in the posttest.

Dimension	COVID-19/Not COVID-19	Average Range
A1. Professional_commitment	COVID-19	142.42
	No COVID-19	107.97
A2. Digital_Resources	COVID-19	136.34
	No COVID-19	116.73
A3. Digital_Pedagogy	COVID-19	148.38
	No COVID-19	113.79
A4. Evaluation	COVID-19	173.89
	No COVID-19	143.95
A5. Empower	COVID-19	145.58
	No COVID-19	117.83
A6. Facilitate_CD	COVID-19	137.31
	No COVID-19	129.75
General	COVID-19	145.21
	No COVID-19	118.36

**Table 8:** Rho Spearman correlation for the variable time of use of ICT and the areas of competence in group A and group B.

Rho Spearman Correlation for the Variable Time of Use of ICT and the Areas of Competence										
				A1.	A2.	A3.	A4.	A5.	A6.	General
Spearman’s Rho Group A	How long have you been using technology as an educational tool?	Coefficient correlation	1.000	0.039	0.083	0.045	0.011	0.056	0.036	0.057
		Sig. (bilateral)		0.525	0.180	0.467	0.853	0.370	0.566	0.356
		N	263	263	263	263	263	263	263	263
				A1.	A2.	A3.	A4.	A5.	A6.	General
Spearman’s Rho Group B	How long have you been using technology as an educational tool?	Coefficient correlation	1.000	0.222 **	0.199 **	0.303 **	0.222 **	0.281 **	0.352 **	0.320 **
		Sig. (bilateral)		0.000	0.001	0.000	0.000	0.000	0.000	0.000
		N	296	296	296	296	296	296	296	296

**. The correlation is significant at the 0.01 level (bilateral).

## Data Availability

Not applicable.

## Supplementary Materials

The following are available online at https://www.mdpi.com/article/10.3390/ijerph18094756/s1, Table S1: «DigCompEdu Check-In» adaptation.
