# Supplementary material for: The Pandemic and Changes in the Self-Perception of Teacher Digital Competences of Infant Grade Students: A Cross Sectional Study
Source: Int J Environ Res Public Health. 2021 Apr 29;18(9):4756. doi: 10.3390/ijerph18094756 (PMC8124795; doi:10.3390/ijerph18094756)
Supplement: Supplementary file 1 [file ijerph-18-04756-s001.zip › ijerph-1164225-supplementary.pdf]

Supplementary Material

Table S1. «DigCompEdu Check-In» adaptation

Competence Area	Competence	Item	Indicator	Level
1. Professional commitment	A. Organizational communication	I systematically use different digital channels to enhance communication with students and fellow academics e.g., emails, blogs, the department's website, Apps	I rarely use digital communication channels	A1
			I use basic digital communication channels, e.g. e-mail	A2
			I combine different communication channels, e.g., e-mail and class blog or the daprtment's website	B1
			I systematically select, adjust and combine different digital solutions to communicate effectively	B2
			I reflect on, discuss and proactively develop my communication strategies	C1
	B. Professional collaboration	I use digital technologies to work together with colleagues inside and outside my educational organization	I rarely have the opportunity to collaborate with other academics	A1
			Sometimes I exchange materials with colleagues, e.g., via e-mail	A2
			Among colleagues, we work together in collaborative environments or use shared drives	B1
			I exchange ideas and materials, also with academics outside my organization, e.g., in an online professional network	B2
			I jointly create materials with other academics in an online network	C1
	C. Reflective practice	I actively develop my digital teaching skills	I rarely have the time to work on my digital teaching skills	A1

Competence Area	Competence	Item	Indicator	Level
			I improve my skills through reflection and experimentation	A2
			I use a range of resources to develop my digital teaching skills	B1
			I discuss with peers how to use digital technologies to innovate and improve educational practice	B2
			I help colleagues in developing their digital teaching strategies.	C1
	D. Digital training	I participate in online training opportunities e.g., online courses, MOOCs, webinars, virutal conferences…	This is a new area that I have not yet considered	A1
			Not yet, but I am definitely interested	A2
			I have participated in online training once or twice	B1
			I have tried out various different online training opportunities	B2
			I frequently participate in all kinds of online training	C1
2. Digital Resources	A. Selection	I use different internet sites and search strategies to find and select a range of different digital resources	I only rarely use the internet to find resources	A1
			I use search engines and resource platforms to find relevant resources	A2
			I evaluate and select resources on the basis of their suitability for my learner group	B1
			I compare resources using a range of relevant criteria, e.g., reliability, quality, fit, design, interactivity, appeal	B2
			I advise colleagues on suitable resources and search strategies	C1
			I do not create my own digital resources	A1

Competence Area	Competence	Item	Indicator	Level
	B. Creation and modification	I create my own digital resources and modify existing ones to adapt them to my needs	I do create lecture notes or reading lists with a computer, but then I print them	A2
			I create digital presentations, but not much more	B1
			I create and modify different types of resources	B2
			I set up and adapt complex, interactive resources	C1
	C. Administration, exchange and protection	I effectively protect sensitive content, e.g. exams, students' grades, personal data	I do not need to do that, because the department takes care of this	A1
			I avoid storing personal data electronically	A2
			I protect some personal data	B1
			I password protect files with personal data	B2
			I comprehensively protect personal data, e.g., combining hard-to-guess passwords with encryption and frequent software updates	C1
3. Digital pedagogy	A. Teaching and Learning	I carefully consider how, when and why to use digital technologies in teaching, to ensure that they are used with added value	I do not or only rarely use technology in class	A1
			I make basic use of available equipment, e.g., digital whiteboards or projectors	A2
			I use a variety of digital strategies in my teaching	B1
			I use digital tools to systematically enhance teaching	B2
			I use digital tools to implement innovative pedagogic strategies	C1
	B. Guide	I monitor my students' activities and interactions in the collaborative online environments we use	I do not use digital environments with my students	A1
			I do not monitor student activity in the online environments we use	A2
			I occasionally check on them and their discussions	B1
			I regularly monitor and analyse my students' online activity	B2

Competence Area	Competence	Item	Indicator	Level
			I regularly intervene with motivating or corrective comments	C1
	C. Collaborative learning	When my students work in groups or teams, they use digital technologies to acquire and document evidence	My students do not work in groups	A1
			It is not possible for me to integrate digital technologies into group work	A2
			I encourage students working in groups to search for information online or to present their results in digital format	B1
			I require students working in teams to use the internet to find information and present their results in a digital format	B2
			My students exchange evidence and jointly create knowledge in a collaborative online space	C1
	D. Self-directed learning	I use digital technologies to allow students to plan, document and monitor their learning themselves<br>e.g., quizzes for self-assessment, ePortfolios for documentation and showcasing, online diaries/blogs for reflection...	Not possible in my work environment	A1
			My students do reflect on their learning, but not with digital technologies	A2
			Sometimes I use, for example, quizzes for self-assessment	B1
			I use a variety of digital tools to allow learners to plan, document or reflect on their learning	B2
			I systematically integrate different digital tools to allow learners to plan, monitor and reflect on their progress	C1
4. Evaluation	A. Assessment strategies	I use digital assessment formats to monitor student progress	I do not monitor students' progress	A1
			I do monitor students' progress regularly, but not with digital means	A2
			Sometimes I use a digital tool, e.g., a quiz, to check on students' progress	B1

Competence Area	Competence	Item	Indicator	Level
			I use a variety of digital tools to monitor student progress	B2
			I systematically use a variety of digital tools to monitor student progress	C1
	B. Analysis of evidence and evidence	I analyse all data available to me to timely identify students who need additional support<br>"Data" includes: students' engagement, performance, grades, attendance; activities and social interactions in (online) environments;<br>"Students who need additional support" are: students who are at risk of dropping out or underperforming; students who have learning disorders or specific learning needs, students who lack transversal skills, e.g., social, verbal or study skills.	These data are not available and/or it is not my responsibility to analyse them	A1
			I only analyse academically relevant data, e.g., performance and grades	A2
			I also consider data on student activity and behaviour to identify students who need additional support	B1
			I regularly screen all available evidence to identify students who need additional support	B2
			I systematically analyse data and intervene in a timely manner	C1
	C. Feedback and planning	I use digital technologies to provide effective feedback	Feedback is not necessary in my work environment	A1
			I do provide feedback to students, but not in digital format	A2
			Sometimes I use digital ways of providing feedback, e.g., automatic scores in online quizzes, comments or "likes" in online environments	B1
			I use a variety of digital ways of providing feedback	B2
			I systematically use digital approaches to provide feedback	C1

Competence Area	Competence	Item	Indicator	Level
5. Empower	A. Accessibility and inclusion	When I create digital assignments for students I consider and address potential digital problems	I do not create digital assignments	A1
			My students do not have problems with using digital technology	A2
			I adapt the task so as to minimize difficulties	B1
			I discuss possible obstacles with students and outline solutions	B2
			I allow for variety, e.g. I adapt the task, discuss solutions and provide alternative ways for completing the task	C1
	B. Differentiation and customization	I use digital technologies to offer students personalised learning opportunities e.g. I give different students different digital tasks to address individual learning needs, preferences and interests	In my work environment, all students are required to do the same activities, irrespective of their level	A1
			I do provide students with recommendations for additional resources	A2
			I provide optional digital activities for those who are advanced or lagging behind	B1
			Whenever possible, I use digital technologies to offer differentiated learning opportunities	B2
			I systematically adapt my teaching to link to students' individual learning needs, preferences and interests	C1
	C. Active participation of students	I use digital technologies for students to actively participate in classes	In my work environment it is not possible to actively involve students in class	A1
			I do involve students actively, but not with digital technologies	A2
			When instructing, I use motivating stimuli, e.g., videos, animations, cartoons	B1
			My students engage with digital media in my classes, e.g., electronic worksheets, games, quizzes	B2

Competence Area	Competence	Item	Indicator	Level
			My students systematically use digital technologies to investigate, discuss and create knowledge	C1
6. Facilitating Learners' Digital Competence	A. Information and media literacy	I teach students how to assess the reliability of information and to identify misinformation and bias	This is not possible in my subject or work environment	A1
			I occasionally remind them that not all online information is reliable	A2
			I teach them how to discern reliable and unreliable sources	B1
			I discuss with students how to verify the accuracy of information	B2
			We comprehensively discuss how information is generated and can be distorted	C1
	B. Communication and digital collaboration	I set up assignments which require students to use digital means to communicate and collaborate with each other or with an outside audience	This is not possible in my subject or work environment	A1
			Only on rare occasions are my students required to communicate or collaborate online	A2
			My students use digital communication and cooperation mainly among each other	B1
			My students use digital ways to communicate and to cooperate with each other and with an external audience	B2
			I systematically set assignments that allow students to slowly expand their skills	C1
	C. Creation of digital content	I set up assignments which require students to create digital content e.g. videos, audios, photos, digital presentations, blogs, wikis...	This is not possible in my subject or work environment	A1
			This is difficult to implement with my students	A2
			Sometimes, for diversion and motivation	B1

Competence Area	Competence	Item	Indicator	Level
			My students create digital content as integral part of their study	B2
			This is an integral part of their learning and I systematically increase the level of difficulty to further develop their skills	C1
	D. Responsible use	I teach students how to behave safely and responsibly online	This is not possible in my subject or work environment	A1
			I inform them that they have to be careful with relaying personal information online	A2
			I explain the basic rules for safely and responsibly acting in online environments	B1
			We discuss and agree on rules of conduct	B2
			I systematically develop my students' use of social rules in the different digital environments we use	C1
	E. Digital troubleshooting	I encourage students to use digital technologies creatively to solve concrete problems e.g., to overcome obstacles or challenges emerging in the learning process	This is not possible with my students, in my work environment	A1
			I rarely have the opportunity to foster students' digital problem solving	A2
			Occasionally, whenever an opportunity arises	B1
			We often experiment with technological solutions to problems	B2
			I systematically integrate opportunities for creative digital problem solving	C1
